# Possible drivers of self-harm severity - relative contributions of post-traumatic stress and levels of personality functioning – results from the Extreme Challenges project

**DOI:** 10.3389/fpsyt.2026.1911977

**Published:** 2026-09-21

**Authors:** Elfrida Hartveit Kvarstein, Tuva Langjord, Tone Bovim, Tore Buer Christensen, Ingeborg Ulltveit-Moe Eikenæs, Oddbjørn Hove, Arvid Nikolai Kildahl, Erlend Mork, Kristine Høst Poulsen, Line Indrevoll Stänicke, Thea Schønning, Tone Tveit, Fredrik Walby, Geir Pedersen, Øyvind Urnes

**Affiliations:** 1 Division of Mental Health and Addiction, Oslo University Hospital, Oslo, Norway; 2 Institute of Clinical Medicine, University of Oslo, Oslo, Norway; 3 Institute of Psychology, University of Oslo, Oslo, Norway; 4 Regional Centre - Violence, Trauma and Suicide Prevention, Oslo, Norway; 5 Division of Medicine, Oslo University Hospital, Oslo, Norway; 6 Department of Adult Psychiatry, Sørlandet Hospital, Arendal, Norway; 7 Department of Research and Innovation, Helse Fonna Health Trust, Haugesund, Norway; 8 Department of Rare Diseases, Oslo University Hospital, Oslo, Norway; 9 Psychiatry East, Central and West Zealand Hospital, Roskilde, Denmark; 10 Department of Psychology, University of Southern Denmark, Odense, Denmark; 11 Lovisenberg Hospital, Oslo, Norway; 12 Division of Psychiatry, Haukeland University Hospital, Bergen, Norway

**Keywords:** personality disorder, personality functioning, post-traumatic stress disorder, self-harm, self-harm severity

## Abstract

**Background:**

Self-harm (SH) is common, but its severity varies. Severe cases may involve extensive hospitalization and high suicide risk. Although SH is associated with post-traumatic stress disorder (PTSD), personality disorder (PD), and complex cases of PD-PTSD comorbidity, factors related to SH severity are sparsely researched.

**Aims:**

The study aims to investigate how SH severity relates to PTSD symptoms and different domains of personality functioning. The study also aims to explore indirect associations of interpersonal functioning, PTSD symptoms and SH severity.

**Methods:**

In a multicenter cross-sectional design, the study included outpatients admitted for personality disorder treatment (*n* = 361) and psychiatric inpatients (*n* = 42) with frequent (>5) or long (>4 weeks) admissions in the previous year due to SH (*N*_total_ = 403, period 2019–2022). Patient self-reports included current (last 6 months) SH and suicide attempts, PTSD symptoms, and self and interpersonal domains of personality functioning.

**Results:**

More severe PTSD symptoms and poorer self-functioning (self-control and identity integration) were significantly and independently associated with greater SH severity. The association between PTSD symptoms and increasing SH severity was partially mediated by greater impairment of self-control and identity integration. Interpersonal domains were not associated with SH severity.

**Conclusion:**

This study highlights post-traumatic stress and personality problems as common clusters of symptoms among patients with increasingly severe SH. Further exploration indicated personality functioning self-domains as potential drivers of the association between post-traumatic symptoms and SH severity. Results support integrated, individualized treatment addressing SH in light of both trauma burden and personality functioning.

## Introduction

Deliberate self-harm (SH) behaviors are frequent in clinical populations, especially in younger adult/adolescent cohorts, but vary in severity, persistence, and intentions (1, 2). The term deliberate SH refers to an intentional act of self-harm with a non-fatal outcome and is irrespective of underlying suicide intention (3, 4). At the extreme end, a small but severely inflicted subgroup develops repeated hospitalizations due to increasingly high-risk SH, which may either be difficult to distinguish from a suicide attempt or coupled with some degree of suicide intention (5). Such extreme SH conditions represent major mental health and health service issues but are nonetheless often not found eligible for available outpatient treatments and often not included in SH research (6–8). Extreme Challenges is a Norwegian clinical, multicenter research project specifically addressing deliberate SH and the lack of studies including severe conditions. Despite the high clinical significance of severe SH development, few have studied possible drivers of SH severity within a broad range of clinically representative patients.

SH severity may relate to a complex interplay of individual capacities, experiences, and environmental context (9). Post-traumatic stress disorder (PTSD) is increasingly associated with SH behaviors, highly relevant among adolescents and young adults (10). It is defined by exposure to an extremely threatening event followed by current trauma-related symptoms (11). Although PTSD is a distinctly defined category, the magnitude of PTSD symptoms may vary. Personality disorders (PDs) are also often associated with SH (12). Among specific PDs (DSM-5), both SH and suicide attempts are among the defining criteria of borderline PD (13–16). However, in clinical samples, severity of condition varies, and PD comorbidity is frequent (17). Patients often present with traits across several categories, among which anxious cluster C disorders, and avoidant PD in particular, are prevalent (18). Research on traditional PD classification has indeed indicated limited validity of the categorical approach (19). As a result, contemporary PD classification represents a move from a categorical system to a dimensional approach with the overall personality functioning capacity as a global defining criterion of PD and its severity (20). Such capacity for personality functioning has been conceptualized within self and interpersonal domains (20, 21). In SH research, emotional regulation is a self-aspect of personality functioning which is often highlighted and emphasized for treatment intervention (22, 23). Interestingly, a former study from the Extreme Challenges project also demonstrated noteworthy problems of social cognition among severe SH inpatients (24). Such findings suggest that both self and relational aspects of personality functioning might have a role in the development of severe SH. Dimensional assessment evaluating an individual’s capacities within both self and interpersonal personality functioning may therefore provide a more nuanced understanding relevant for SH severity and identification of treatment-relevant features. As yet, research on SH and personality disorders is mainly focused on borderline PD, and few have investigated associations with different capacities of personality functioning.

The co-occurrence of PD and PTSD is common (25–28), and the conditions impact each other’s expressions. Impaired personality functioning may render higher risk for traumatic exposures, complicate trauma processing, and thereby contribute to the development of PTSD. Conversely, ongoing PTSD may accentuate personality problems. This is acknowledged in the ICD 11 concept of complex PTSD (29), reflecting more enduring disturbance of personality functioning (affect regulation, self-concept, and relationships) (30). In a study of a population with repeated acute psychiatric hospitalizations, both personality problems of emotional dysregulation and PTSD emerged as independent clinical concerns (31), and previous research from the current project has specifically highlighted the dual PD-PTSD condition as a central aspect of complex, severe, SH conditions (6). Aspects of personality functioning can be considered the more stable, underlying qualities, and impairments may therefore impact a patient’s drive for self-harm. Interestingly, recent treatment developments for populations with personality problems and high traumatic burden emphasize a stronger focus on interpersonal capacities (32).

Deliberate SH behaviors are most often non-suicidal actions. As a consequence, research has often been limited by the definition of SH as “non-suicidal self-injury” (NSSI) (33, 34). However, the risk of completed suicide among people who SH is noteworthy (35), and in a study of 600 self-harming adolescents, significant risk of suicide was detected in approximately 50% (36). Moreover, in a study of suicide completers with PD, the majority had a former history of extensive emergency health service use and repeated SH (37). More recent research on young adult inpatients with severe SH has also indicated a developmental history from moderate SH starting in adolescence, and over time, increasing SH frequency, suicidal intent, and repeated hospital admissions (6). The combination of frequent SH and occurrence of suicide attempts can thus be seen as a marker of severity in terms of enhanced risk. However, few studies have examined SH severity in terms of increasingly repetitive SH and occurrence of concurrent suicide attempts. The clinical impact of early identification of factors associated with more severe SH developments is considerable, including possible prevention of fatal incidents and alleviation of substantial personal and societal burden associated with longstanding severe SH conditions (38, 39).

Addressing the underrepresentation of SH research also incorporating the most severe SH conditions, the present study investigates deliberate SH as “*intentional non-fatal SH irrespective of suicidal intent*,” a definition in accordance with other research featuring challenges of adverse developments present in clinical samples (40, 41), and ranges the combination of current highly repetitive SH and non-fatal suicide attempts as the most severe SH situation. The present study sample is large, characterized by varying presence and severity of SH, personality functioning, and traumatic burden, and is recruited within relevant, regular mental health services.

### Objectives

The study aims to investigate 1) relations between SH severity and current symptoms of PTSD and 2) relations between SH severity and problems of personality functioning. In line with previous research, we hypothesize that SH severity will be significantly associated with 1) PTSD symptoms and 2) both self and interpersonal domains of personality functioning. In an additional investigation, addressing the noted assumptions of personality functioning representing the more stable, underlying quality, we aim to 3) explore indirect associations in a mediation model including personality functioning and PTSD symptoms in relation to SH severity. We hypothesize that the relation between PTSD symptoms and SH severity will be associated with patients’ level of personality functioning.

## Methods

### Study design

The present study has a cross-sectional, multisite, exploratory design including patients across Norwegian health regions.

### Setting

In order to capture a target group of patients that could represent variation in SH, impairment of personality functioning, and traumatic background, patients were recruited from two different contexts: 361 were outpatients from 13 PD treatment units and 42 were inpatients from 12 psychiatric hospitals.

### Participants

Outpatient data were retrieved retrospectively (2022) from the quality register of the Norwegian Network for Personality Disorders (NNPD) (42) and included patients with baseline assessment of the frequency of SH and non-fatal suicide attempts (SAs) for the last 6 months and patient-reported personality functioning and current post-traumatic symptom burden. NNPD is a cross-regional quality and research collaboration of treatment units within specialized mental healthcare, providing specialized PD treatment for patients with PD and personality difficulties of varying severity and types. Inpatient inclusion criteria were frequent psychiatric hospital stays during the last 12 months (defined as >5 admissions) and/or long hospital stays (>4 weeks duration) due to severe risk of SH and/or SA, irrespective of frequency, type, or SH/SA severity (6).

### Sample size

The total study sample counted 403 individuals, all recruited from adult mental health services (18–65 years) with assessments of SH, non-fatal SA, personality functioning, and trauma symptoms performed in the period 2019–2022.

### Variables

Assessment of SH and non-fatal SA was based on items originally developed as patient self-reports in NNPD and included SH and SA assessment recording over the lifetime (ever) and during the last 6 months (current; answer options: yes/no, and if yes, age at first occurrence and frequency of SH based on specified categories) (6). The specified categories of SH frequency included monthly or less, weekly, or daily. A composite variable termed SH severity was computed by combining variables on SH frequency and the occurrence of non-fatal SA during the last 6 months. This variable was measured on a scale from 0 to 7, with the following scores: 0, no SH/SA during the last 6 months; 1, no SA, but SH occurring monthly or less; 2, no SA but weekly SH; 3, no SA but daily SH; 4, SA during the last 6 months but no SH; 5, SA and SH occurring monthly or less; 6, SA and weekly SH; and 7, SA and daily SH. Validity of the composite variable SH severity was tested by correlating SH severity with a patient-reported measure of the level of depressive symptoms—Patient Health Questionnaire, Depression, PHQ-9 (43, 44). In the study sample of *N* = 403 patients, the Pearson correlation for the PHQ-9 sum score was *r* = 0.338, indicating a moderately positive linear relationship (*p* < 0.001) and shared variance, *r*^2^ = 11.4%. The correlation between the SH severity variable and the specific PHQ-9 item on suicide ideation (item 9) was highly positive (*r* = 0.443, *p* < 0.001) with shared variance, *r*^2^ = 19.6%.

PTSD symptoms and personality functioning included the following patient self-reports:

1. PTSD Checklist for DSM-5 (PTSD-CL-5) (45) (PCL-5) (4) includes an initial screening for traumatic experiences (severe illness, physical assault, sexual assault, childhood physical maltreatment, war, accident, natural disaster, others) and then a 20-item assessment of the severity of current PTSD symptoms, rated on a 0–4 scale (intrusion: 5 items, avoidance: 2 items, negative cognitions and mood: 7 items, arousal and reactivity: 6 items). A sum score of 33 or higher is considered to indicate possible PTSD. Psychometric validations include a study based on the Norwegian versions of the PCL-5 used in the present study (46). In the present study, a PCL-5 score of 0 implied either the presence of a traumatic experience without PTSD symptoms (*n* = 3) or a negative initial screening for traumatic experiences (*n* = 111).
2. Levels of Personality Functioning–Brief Form (LPFS-BF 2.0) (47) is a 12-item patient self-report based on the Level of Personality Functioning Scale (DSM-5, Section III, Alternative Model of Personality Disorders). Psychometric evaluations include former studies based on Norwegian translations of the instrument used in the present study (48, 49). The present study reports a 0–3 response scale (“very false or often false” to “very true or often true”). Items 1–6 indicate problems of self-functioning and items 7–12 relational problems. A recent Danish study indicated clinical severity of personality dysfunction at a level consistent with personality disorder for scores >14 (50). The LPFS-BF gives an overall sum score as well as scores for the self and interpersonal subdomains. It was included in this study as an overall measure frequently reported in contemporary research on personality disorder and included in the International Consortium for Health Outcomes Measurement for personality disorder (51).
3. The Severity Indices of Personality Problems – Short Form (SIPP-SF) is a 60-item patient self-report reflective of central self and interpersonal aspects of personality functioning (52). It was developed from the original, well-established SIPP 118 (53); its evaluations also include studies based on Norwegian translations of the instrument used in the present study (54, 55). The SIPP was originally designed with the explicit aim of covering core components of (mal)adaptive personality functioning and has been frequently used in Norway as reflected in several clinical, multicenter research projects (18, 56). SIPP-SF was included in the present study as it could reflect more specific aspects of personality functioning, thus supplementing the more global LPFS-BF. SIPP-SF factor structure indicates the corresponding five domains as the SIPP-118 (57). SIPP-SF is rated on a 1–4 scale from “fully disagree” (1) to “fully agree” (4). The domains include two self-aspects: *self-control* (capacity to control emotions, aggression, and impulsive reactions or behaviors) and *identity integration* (capacity to understand own mental states, motives, and identity; experience self-worth, enjoyment, and purpose), and three interpersonal aspects: *social concordance* (the ability to value someone’s identity, cooperate, accept interpersonal differences, and withhold aggressive impulses toward others), *relational capacity* (capacity to communicate thoughts, feelings and affections to others; become mutually attached; feel cared about; and experience long-term relationship), and *responsibility* (ability for enduring agency, setting goals, and achieving them in line with the expectations of others, keeping rules, agreements, and commitments). In this study, we report *t*-scores (scores < 40 indicate deviation from Norwegian population norms).

### Other measurements

Additional information specifying childhood trauma experience was assessed by the Childhood Trauma Questionnaire (CTQ) (58). CTQ is a 28-item self-report questionnaire (1–5 scale) assessing the presence and severity of emotional neglect, emotional abuse, physical neglect, physical abuse, and sexual abuse. Diagnoses of PD were based on the Structured Clinical Interview for DSM-5 Personality Disorders (SCID-5-PD) (59). Interviews were performed by experienced clinicians with relevant training, as detailed in a former study (6). In the present study, fulfilled diagnostic categories and the total number of SCID-5-PD criteria are presented as descriptive background information. For the evaluation of other mental health disorders, the M.I.N.I. International Neuropsychiatric Interview was recommended in the outpatient cohort and used systematically for all patients in the inpatient cohort (MINI) (60). Social functioning was rated using the Work and Social Adjustment Scale (WSAS) (61), a 5-item patient self-report (0–8 scale; 0: no impairment, 8: extreme impairment), assessing work or study functioning, home management, social leisure activities, private leisure activities, and close relationships, with clinically relevant impairment indicated by a WSAS sum score >15 (62, 63). Other mental health symptom distress was indicated by patient self-reports: the Patient Health Questionnaire, Depression (PHQ-9, 9 items rated on a 0–3 scale) (43, 44) and the Generalized Anxiety Disorder (GAD-7, 7 items, 0–3 response scale) (64). For both measures, sum scores ≥10 indicate clinically relevant symptoms (65–67).

### Ethics

Participation required informed, written consent. Procedures for the inpatient data collection (inpatient cohort) were approved by the Norwegian Regional Ethics Committee (REK; 2018/1124/REK Sør-Øst). All data collection procedures were approved by the local Data Protection Officer at each contributing hospital/treatment unit. The quality register of the Network for Personality Disorders (outpatient cohort) is based on anonymous clinical data, and formal approvals from the Norwegian State Data Inspectorate and Medical Research and Ethics Committee were not required. Security procedures for the quality register were approved by the Data Protection Officer at the responsible center (Oslo University Hospital). The project Extreme Challenges has preregistration in ClinicalTrials.gov (NCT03768674). Procedures are as presented in former publications within the Extreme Challenges project (6).

### Statistical methods

Statistical analyses were performed using IBM SPSS version 30, including the SPSS PROCESS MAKRO version 5.0. Statistical analyses were based on linear regression models following the principles of maximum likelihood (mixed models, SPSS) (68) and included the total study sample (*N* = 403). Regression models achieved convergence and demonstrated the best model fit, with fixed effects and no specified random effects. The dependent variable was SH severity in all models, measured on a seven-level scale. Considering the large sample size (>400 patients), the skewness (1.53, SE: 0.12) and kurtosis (0.94, SE: 0.24) of the LMM-based residuals for the SH severity variable fell within acceptable thresholds for the assumption of a normal distribution.

Investigation of possible predictors (independent variables) included PCL-5 sum score, LPFS-BF self and interpersonal domains, and all five SIPP-SF domains. The strength of the models with predictor interactions was evaluated according to the significance of the fixed effect (*p* < 0.05), improvement of the model fit [Akaike information criterion (AIC), where a smaller value is better, ΔAIC], and change in marginal pseudo-*R*^2^—a measure that indicates the proportion of variance explained by fixed effects. To address our research aims, analyses were conducted stepwise: first, separate models were fitted for the PCL-5, LPFS-BF, and SIPP-SF domains, followed by models for the LPFS-BF domains and PCL-5 and for the SIPP-SF domains and PCL-5. The models are presented in **Table 1**. As considerable variation could be associated with differences between inpatients and outpatients, the models finally included a variable differentiating between outpatients and inpatients. As a large proportion had high depression scores (PHQ-9), control analyses including PHQ-9 were also performed.

**Table 1:** Associations between SH severity, personality functioning, and traumatic distress.

N = 403		Fixed effect		Pseudo-R^2^	Model fit
*Dependent variable*	*Independent variable*	*Estimate (SE)*	*p*	*Marginal*	*AIC*
SH severity		1.32 (0.1)	<0.001	0	1,710
Separate models					
SH severity	PCL-5 sum score	0.02 (0.004)	<0.001	0.08	1,679
SH severity	LPFS-BF sum score	0.05 (0.02)	<0.001	0.03	1,701
SH severity	LPFS-BF sum self	0.12 (0.03)	<0.001	0.04	1,697
	LPFS-BF sum other		ns		
SH severity	T SIPP-SF - self-control	−0.04 (0.01)	<0.001	0.13	1,665
	T SIPP-SF - identity	−0.05 (0.01)	<0.001		
	T SIPP-SF - social concordance		*ns*		
	T SIPP-SF - relation capacity		*ns*		
	T SIPP-SF - responsibility		*ns*		
Model with LPFS-BF and PTSD symptoms					
SH severity	LPFS-sum self	0.09 (0.03)	0.006	0.10	1,675
	LPFS-sum other		ns		
	PCL-5 sum score	0.02 (0.004)	<0.001		
Model with SIPP-SF and PTSD symptoms					
SH severity	T SIPP-SF - self-control	−0.03 (0.01)	<0.001	0.16	1,652
	T SIPP-SF - identity	−0.04 (0.01)	<0.001		
	T SIPP-SF - social concordance		*ns*		
	T SIPP-SF - relation capacity		*ns*		
	T SIPP-SF - responsibility		*ns*		
	PCL-5 sum score	0.02 (0.004)	<0.001		
*Models differentiating* (Δ) inpatients from outpatients					
SH severity	Inpatient cohort	4.45 (0.3)		0.28	1,578
	Δ Outpatient - inpatient cohort	−3.49 (0.3)	<0.001		
SH severity	Inpatient cohort * PCL-5 sum-score	0.07 (0.005)	<0.001	0.30	1,572
SH severity	Inpatient cohort * self-control	−0.03 (0.008)	<0.001	0.37	1,537
	Inpatient cohort * identity	−0.03 (0.01)	0.008		
	Inpatient cohort * social concordance		*ns*		
	Inpatient cohort * relation capacity		*ns*		
	Inpatient cohort * responsibility		*ns*		
SH severity	Inpatient cohort * self-control	−0.03 (0.01)	0.001	0.37	1,538
	Inpatient cohort * identity	−0.02 (0.01)	0.01		
	Inpatient cohort * social concordance		*ns*		
	Inpatient cohort * relation capacity		*ns*		
	Inpatient cohort * responsibility		*ns*		
	Inpatient cohort * PCL-5 sum score		*ns*		

Mean estimates with standard errors (SE) based on different linear models of the associations between self-harm (SH) severity and 1) PTSD, 2) personality functioning (LPFS-BF and SIPP SF subscales), 3) models combining PTSD and measures of personality functioning, and 4) models including differences (Δ) between outpatients and inpatients and model-based estimations for the inpatient cohort.

Indications of model fit are indicated by Akaike information criterion (AIC, smaller is better), and model-based explained variation by estimations of marginal *p*seudo-R^2^.

The additional investigation of indirect effects (mediator analyses) explored the relationship between traumatic symptom burden (PCL-5 score = X), personality functioning (SIPP-SF - self-control = M1, SIPP-SF Identity Integration = M2), and SH severity (Y). The mediation analyses were performed with bootstrapping (5,000 resamples) (69). Analyses include path A (X → M), path B (M → Y while controlling for X), and path C′, the direct effect (X → Y while controlling for M). The indirect effect of X on Y (mediation) is the product of coefficients for path A and path B. Mediation is statistically significant if the bootstrapped confidence intervals do not contain zero; mediation is partial if both the indirect and the direct effects are significant, and it is complete if only the indirect effect is significant. The proportion mediated can be indicated as follows: Indirect effect/Total effect (sum of indirect and direct effect) × 100.

## Results

### Descriptive data

#### Self-harm severity

A total of 74% reported former SH and 50% reported former SA. SH/SA started before 18 years of age for the majority (SH: 80%, SA: 62%), and 68% reported having their first contact with mental health services before 18 years of age. Former experience of admission to a psychiatric hospital was reported by 38%. **Table 2** demonstrates current SH severity (the last 6 months) in the study sample (*N* = 403) and differentiates between severe inpatients and outpatients admitted for PD treatment. One-third of the patients (33%) in the outpatient sample reported SH, but not suicide attempts during the last 6 months, and 11% reported a combination of SH and suicide attempts. In the inpatient sample, 19% reported SH, but not suicide attempts during the last 6 months, whereas approximately two-thirds (67%) reported a combination of SH and suicide attempts. In the total study sample, 47% reported current SH, and 15% reported a combination of SH and SA, with a mean SH severity score of 1.32 (SD 2.0, median 0, range 7). The difference in SH severity for the inpatient and outpatient subsamples was highly significant [mean estimated SH score for inpatients: 4.45 (0.3), mean estimated cohort difference: 3.49, SE 0.3, *p* < 0.001, 28% explained SH severity variation, ΔAIC = 132].

**Table 2:** Self-harm (SH) and suicide attempts (SA).

SH severity		Outpatient		Inpatient		Total study sample	
		*n*	%	*n*	%	*N*	%
0	No SH/SA for the last 6 months	209	58	3	7	212	53
1	Monthly or less SH, no SA	85	24	5	12	90	22
2	Weekly SH, no SA	27	8	2	5	29	7
3	Daily SH, no SA	2	1	1	2	3	1
4	SA no SH	7	2	3	7	10	3
5	SA and monthly or less SH	15	4	9	21	24	6
6	SA and weekly SH	14	4	15	36	29	7
7	SA and daily SH	2	1	4	10	6	2
Total		361	100	42	100	403	100

The distribution of SH severity in the total study sample and inpatient and outpatient subgroups measured by the applied SH severity scalewith seven levels indicating increasing repetitions and occurrence of suicide attempts.

#### Trauma, personality, and psychosocial status

**Table 3** demonstrates PD and trauma status. In the total sample, 72% (*n* = 292) reported some kind of former trauma experience on the PCL-5 screening and 48% had PCL-5 sum scores indicating current PTSD. Among patients with PCL-5 sum scores >33, screening of trauma experiences included severe illness (33%), physical assault (41%), sexual assault (54%), childhood physical maltreatment (28%), serious accident (25%), war (2%), natural disaster (3%), and others (54%). Specification of childhood trauma (CTQ) among patients with PCL-5 sum scores >33 indicated moderate to severe physical abuse (23%), sexual abuse (31%), emotional abuse (59%), emotional neglect (65%), and physical neglect (43%). All five CTQ aspects were scored significantly higher among patients with current PCL-5 sum scores >33 [*p* < 0.001, effect sizes (Cohen’s *d*) ranged 0.5–0.7, largest for emotional abuse].

**Table 3:** Descriptive background: trauma, personality, and psychosocial status.

Descriptive background: trauma, personality, and psychosocial status					
	Total sample		Inpatient sample		Outpatients versus inpatients
Diagnostic assessment	%	Mean (SD)	%	Mean (SD)	*p*
No PD	21		9		
Cluster A	12		17		
Cluster B	47		69		
Cluster C	53		69		
Total SCID-5-PD criteria		12.3 (6.2)		14.6 (7.1)	0.02
Other mental disorders (number)		1.8 (1.9)		4.9 (2.4)	<0.001
Trauma experience (PCL-5)	%		%		
Severe illness	24		45		<0.05
Physical assault	25		48		<0.05
Sexual abuse	29		78		<0.05
Childhood physical maltreatment	16		35		<0.05
Other	55		80		<0.05
	% > cutoff		% > cutoff		
PCL-5 sum score	48	28.0 (23.0)	76	50 (20.4)	<0.001
CTQ total		5.0 (3.9)		6.3 (4.5)	0.03
LPFS-BF 2.0	82	19.2 (6.4)	69	17.4 (7.3)	*ns*
T SIPP-SF self-control	71	30.4 (14.7)	64	30.2 (16.1)	*ns*
T SIPP-SF identity	95	20.8 (11)	95	15.9 (11.6)	0.002
T SIPP-SF relational functioning	72	33.5 (10.7)	64	35.0 (12.9)	*ns*
T SIPP-SF social concordance	47	40.2 (14.9)	26	46.4 (14.9)	0.04
T SIPP-SF responsibility	67	33.3 (13.6)	62	35.2 (13.6)	*ns*
Depression symptoms (PHQ-9)	95	18.4 (5.2)	98	22.4 (3.9)	<0.001
Anxiety symptoms (GAD 7)	82	13.7 (4.4)	88	15.3 (4.6)	0.02
Social functioning (WSAS)	89	23.6 (7.4)	98	26.5 (5.9)	0.03

**Table 3** demonstrates the distribution of diagnostic groups in % and means with standard deviation (SD) based on observer-rated diagnostic data and other clinical information based om patient self-report with mean scores and % over clinical cutoffs.

Instruments with full names and acronyms and the applied clinical cutoff levels are presented in the Methods section. For categorical variables, significant differences between outpatients and inpatients are indicated by *p* < 0.05 (chi-square test); for continuous variables, *p* > 0.05 is indicated by *ns*; and statistics are based on multivariate analysis of variance.

The majority in the total sample qualified for a diagnosis of PD (79%), mainly within clusters B and C. The mean LPFS-BF score indicated impaired personality functioning on a moderate severity level. Poorest functioning was reported for the SIPP-SF self-domain, identity integration. Most domains of SIPP-SF had poorer scores among patients who also had a PCL-5 sum score >33 (both SIPP-SF self-domains: *p* < 0.001, Cohen’s *d* = 0.5; SIPP-SF-social concordance, *p* < 0.006, Cohen’s *d* = 0.3; SIPP-SF relational functioning, *p* < 0.01, Cohen’s *d* = 0.3).

**Table 3** demonstrates general psychosocial status. In the total sample, work and social functioning was clearly impaired, comorbidity was frequent, and 48% qualified for two or more mental health disorders other than PD. Patient self-report of symptoms indicated considerable mental distress.

### Main results

**Table 1** presents the linear regression estimates. Correlations among key variables are given in the **Supplementary File** (**Supplementary Table 1**).

#### Self-harm severity and PTSD symptoms

In separate SH severity models, more severe SH was significantly associated with higher scores of PCL-5 sum score. Trends remained in the models adding differentiation between inpatients and outpatients and in the models controlling for symptoms of depression.

#### Self-harm severity and personality functioning

In separate SH severity models, more severe SH was significantly associated with an overall increase of LPFS-FS sum score and, specifically, more severe problems within self-domains of personality functioning (LPFS-BF self-domain and SIPP-SF self-control and identity integration). SH severity was not significantly associated with the relational domains of LPFS-BF or SIPP-SF in separate models nor when combining all relational domains in one model (all *p* > 0.05). SH severity models investigating LPFS-BF sum score had a poorer fit compared with models specifying self and other subdomains and models further specifying SIPP-SF domains (**Table 1**). Trends remained in the models adding differentiation between inpatients and outpatients and in the models controlling for symptoms of depression.

#### Self-harm severity, personality functioning, and PTSD symptoms

In the SH severity model investigating PCL-5 and both domains of LPFS-BF together and in the SH model investigating PCL-5 and the five domains of SIPP-SF together, associations remained highly significant for both PCL-5 and the self domains of LPFS-BF and SIPP-SF (**Table 1**). In the models with the five SIPP-SF domains, adding differentiation between inpatients and outpatients, PCL-5 sum score had no significant independent effect in the inpatient subsample, and the inclusion of PCL-5 did not further improve the model fit nor add explained variance (**Table 1**). Trends remained in the models controlling for symptoms of depression.

We further investigated the potential role of personality functioning as a possible mediator of the relationship between post-traumatic symptom burden (PCL-5) and SH severity. Estimates for the two SIPP-SF self-domains are given in **Table 4**. An illustration of the mediation model is given in the **Supplementary File** (**Supplementary Figure 1**). Indirect effects were significant in both models: M1 (self-control), 0.005 (bootstrapped SE: 0.0015; bootstrapped 95% CI [0.0024, 0.0083]) and M2 (identity integration), 0.005 (bootstrapped SE: 0.0014; bootstrapped 95% CI [0.0028, 0.0083]). Overall results indicated a partial mediating effect of these self-functioning aspects, and the proportion mediation was 21% for both models. Further investigation of the three interpersonal domains of SIPP-SF revealed non-significant indirect effects.

**Table 4:** Indirect effects of personality functioning.

*N* = 403	*B* (SE)	*t*	*p*	95% CI	*R* ^2^
Path A (X → M1)	−0.177 (0.029)	−6.1	<0.001	[−0.2, −0.1]	0.084
Path b (M1 → Y)	−0.028 (0.007)	−4.2	<0.001	[−0.04, −0.02]	0.118
Path C′	0.019 (0.004)	4.5	<0.001	[0.01, 0.03]	
Path A (X → M2)	−0.122 (0.022)	−5.4	<0.001	[−0.17, −0.08]	0.070
Path B (M2 → Y)	−0.043(0.009)	−4.9	<0.001	[−0.06, −0.03]	0.131
Path C′	0.019 (0.004)	4.5	<0.001	[0.01, 0.03]	

Estimates from regression models (paths A, B, and C′) of the mediation analyses. *B* indicates the unstandardized beta coefficients (*B*), with standard error (SE) demonstrated for the given pathways with SH severity (Y), PCL-5 sum score (X), and the two SIPP-SF domains self-control (M1) and identity integration (M2). *R*^2^ (coefficient of determination) indicates overall model fit.

## Discussion

This study includes a large clinical sample treated within regular outpatient and inpatient services within specialist mental health services relevant for people with personality problems and, as such, quite uniquely comprises patients with a broad range of severity of functional impairment and varying presence and severity of SH suitable for the stated study aims.

### Key results

In accordance with the stated study objectives. the main findings are summarized as follows:

1. Greater self-harm severity was associated with higher current PTSD symptom levels.
2. Greater self-harm severity was associated with more severe impairment of self-domains of personality functioning.
3. Self-harm severity was not associated with varying impairment of interpersonal personality functioning.
4. Additional investigation revealed a partial mediating effect of self-aspects of personality functioning on the relation between self-harm severity and PTSD symptoms.

### Study sample characteristics

The present study investigated self-harm apparent within mental health services. A striking majority of the total sample reported former experience of self-harm, adolescent SH debut, and extensive contact with mental health services, and half of the sample described former suicide attempts. Moreover, nearly half the total sample reported *current* SH of some form, and 15% a combination of current SH and current suicide attempts. At the extreme end, 10% of the total sample represented an inpatient cohort with currently repeated or lengthy admissions in a psychiatric hospital and significantly more severe self-harm, 45% of this cohort had lasting SH-related physical sequelae (6), and the majority reported a combination of current SH and suicide attempts. These sample characteristics reflect findings from other studies, demonstrating that severe and repetitive SH often entails suicidal intention, suicide attempts, and also risk of completed suicide (6, 70).

### Self-harm severity was associated with PTSD symptoms

A clinical level of PTSD symptoms was reported in half of the sample. Our first main finding demonstrated that higher PTSD symptom burden was significantly associated with more severe self-harm. The result is in accordance with our first hypothesis relating increasing SH severity to PTSD symptom intensity. Although little research has investigated SH severity, the result is as expected considering the available research documenting associations between PTSD, the presence of SH, and other destructive actions (71–73). In our present study, we also found that the association between the intensity of PTSD symptoms and SH severity was mainly independent of the level of personality functioning and the severity of specific personality problems. However, a noteworthy caveat is that this association was not found independent of personality functioning in the inpatient subsample.

### Self-harm severity was associated with aspects of personality functioning

The large majority qualified for PD, and the global measure of personality functioning (LPFS) indicated a mean level of moderate severity. Our second main finding demonstrated that poorer personality functioning, specifically, problems of self-control and identity integration, was strongly associated with more severe self-harm, independent of the presence and severity of PTSD symptoms.

The magnitude of problems of self-control, largely depicting emotional and behavioral dysregulation found in our study sample, corresponded to reports based on a larger clinical sample of outpatients with different PDs (74). Associations between SH and problems of emotional regulation are generally well established (75). Our findings additionally emphasize the dimensional perspective of increasing SH severity relative to the extent of self-control impairment. It should be noted that self-control is not the only aspect of emotional dysregulation. The quality of emotional problems is often heterogeneous in PD samples, and alexithymia and emotional restrictiveness are frequently also part of the picture (76, 77). The present study did not, however, include measures reflecting emotional restrictiveness and possible associations with SH severity. However, such heterogeneity may have been reflected in the overall only moderately enhanced mean score levels of self-control and comparatively small differences between outpatients and inpatients.

In the present study, problems of identity integration were generally frequent, reflecting considerable problems, and increasing problems were significantly associated with more severe SH. Score levels were significantly poorer in the inpatient subgroup. According to the SIPP-SF inventory, the capacity for identity integration represents the experience of stability and purpose in life. Such problems are commonly encountered among patients with PD and form a central and challenging part of the clinical picture closely aligned with the concept of self-agency (78). Other studies have related SH with experiences of low meaning of life and difficulties engaging in goal-directed behavior (75, 79). Our results specifically underline how identity problems, irrespective of comorbid depression or problems of self-control, can be a central aspect of the clinical SH severity picture. The findings thus provide further nuance to the general understanding of SH and existing emphasis on emotional dysregulation as a driving mechanism.

Contrary to our initial hypothesis, we did not find significant associations with SH severity for the interpersonal domains of personality functioning - neither capacity for close relationships nor more general premises of social interaction and flexibility. Such results indicate that the link to SH severity may not be directly related to relational or social capacities. Interpersonal aspects of personality functioning include the capacity and management of close relationships, and in several previous studies, insecure attachment to caregivers has been associated with enhanced rates of SH and also repetitive SH development (80, 81). Interpersonal malfunctioning can also be a developmental feature, and associations between autism spectrum disorders, SH, and enhanced suicide risk are reported (24, 82, 83). However, in a study of patients with autism spectrum disorders, enhanced suicide risk was particularly linked to high levels of alexithymia and symptoms of depression, both of which represent aspects of self-functioning (83). Moreover, maladaptive interpersonal functioning and the lack of social network or capacity for close relationships may indirectly contribute to problems of emotional or behavioral regulation (84, 85). Notably, we found that patients in our study generally indicated quite moderate problems of interpersonal functioning. The evaluation of interpersonal capacities may be limited by the use of patient self-report and the lack of specific measures of autism traits.

### Partial mediating effect of personality functioning

Our additional finding, based on exploratory mediation analyses, confirmed a significant, though small, indirect association between post-traumatic symptom burden and SH severity, through self-related personality functioning. More specifically, self-control and identity integration accounted for part of the association between PTSD symptoms and SH severity. Clinically, these results encourage a focus on personality functioning when assessing severe SH individuals with traumatic burden.

### Treatment conditions for SH patients

A recent systematic review and meta-analysis underscores a high suicide risk among hospital-admitted SH patients and a need for further outpatient treatment after discharge. The study reported that on a global level, only one-third of hospital-admitted SH patients were referred to outpatient psychiatric care, and older adults (>60) were the least likely to receive treatment (86). In our study sample, including patients admitted to outpatient, personality-focused treatment and patients admitted to a psychiatric hospital, 30% of the outpatient sample also reported a previous experience of psychiatric hospital admission (6).

Research has generally pointed to an insufficient evidence base for SH-focused interventions for adolescents (87), although separate studies demonstrate promising effects of structured interventions such as dialectical behavioral therapy (88–90). Among adults, improvements in SH are reported across different specialized approaches addressing borderline PD; however, the evidence base is also considered here as modest (91). In our study sample, the majority (90%) had been admitted for treatment in an outpatient setting for group-based, personality-focused treatment (92). The applied treatments were generally in a long-term (>1 year) psychotherapy format, and available programs included structured treatments such as mentalization-based treatment or schema-focused treatment or psychodynamic group therapy and combinations of individual support, art therapy, or different cognitive or physical training groups. The present study’s scope is limited by its cross-sectional design; however, quite recent studies from the same personality-focused outpatient treatment setting have indicated longitudinal improvements of corresponding self-aspects of personality functioning with effect sizes in the moderate to large range (74, 76). Moreover, a specific study of mentalization-based treatment pointed to considerable remission of SH among poorly functioning patients with borderline PD during treatment (93). Nearly half of the current sample had comorbid PTSD symptoms and PD. In line with other trauma-focused research (94), further study should investigate the longitudinal course within mental health services for patients with PD and high post-traumatic symptom burden (32, 95, 96).

The inpatient subgroup in our multicenter, cross-national study had severe and repetitive SH—often a longstanding situation likely to represent highly vulnerable patients not responding to available treatments or not having treatment available. Several studies have identified gaps in the delivery of health services, a lack of applicable treatment, and limited understanding of severe SH management (7, 8, 97, 98). The effects of repeated, negative treatment experiences for later treatment compliance should not be underestimated, nor should the iatrogenic effects of long-standing regulatory or compulsory treatment strategies (7). Appropriate treatment in more complex or severe SH conditions is thus of vital importance but appears to expand acknowledged treatment recommendations for PD (91) or PTSD (99). Current research points to more individualized identification of patients’ condition and interventions specifically tailoring central aspects of the complex disorder, including cognitive and developmental challenges as well as personality functioning capacities and traumatic stress symptoms (95, 100–103). Our study specifically underlines the relevance of interventions addressing both self-control and identity integration and thus adds to the often-recommended focus on emotional dysregulation.

### Childhood trauma was part of a complex background picture in severe cases

In our sample, background information indicated significant childhood trauma, both physical and psychological. The inpatient sample, in particular, uncovered a combination of severe mental distress, comorbidity of PD and PTSD, together with high levels of childhood trauma (6). Such severe comorbidity is also previously associated with multiple and interpersonal trauma (104). Childhood trauma is generally associated with poorer psychological resilience, rendering greater difficulties in regaining stability after exposure to traumatic stress, a range of negative health outcomes, and higher susceptibility to health-risk behaviors, including SH and suicidal behaviors (105–108). The development from childhood trauma to PTSD has been related to emotional dysregulation, insecure attachment, introversion, and lack of social support (109–111). In our total study sample, a higher proportion reported childhood trauma than current PTSD symptoms, thus indicating sample heterogeneity, where positive resilience factors are likely to be apparent.

### Strengths and limitations

As the study sample is recruited from regular services, it holds high clinical representativity as it reflects the distribution of patients often reported in studies of treatment-seeking patients with self-harm, with a dominance of the female gender, young adult age group, and the presence of personality problems as well as traumatic background experiences (112, 113). However, this distribution may limit generalizability across other clinical, forensic, or general community contexts, genders, and age cohorts. Interestingly, a study of SH within a forensic population also reported higher prevalence among women and younger age groups (114). Clinical conditions not well covered by our study sample include substance use disorders and addiction, developmental and impulsivity control disorders, bipolar disorders, psychoses, and geriatric conditions.

The SH severity scale was constructed for the purpose of this study and has several possible limitations. As a newly constructed measure, results cannot directly compare with other studies. It aimed to capture the suggested severity developments, from no self-harm, single SH incidents, repetitive SH behaviors, and increasing extent of both current SH behaviors and reported suicidal actions. Our SH variable did not incorporate information on the physical severity of the actual SH or suicide acts. We did not record self-destructivity in terms of excessive substance use or eating disorder as self-harm, although such behaviors can have similar self-regulating or self-punishing functions (115–117). A former study of the inpatient cohort did suggest that self-destructivity could have other channels in addition to what was recorded as self-harm. In this cohort, 44% qualified for substance use disorder and 19% had an eating disorder (6). The current study design was pragmatic, balancing available/retrievable data, practical possibilities for operationalization of SH severity with research emphasizing the risk of developing repetitive SH acts and increasing suicide intentions (70, 118, 119).

As a clinical study, the study sample is large (*N* = 403). Although the distribution of the dependent variable can be considered moderately skewed, the values for the residuals of the linear regression analyses were within a generally accepted range for skewness (−2 to +2) and kurtosis (−7 to +7) and in accordance with the requirements for maximum likelihood statistics.

The investigated predictor variables are all based on patient self-report. The value of patient-reported experiences is generally well established. In the present study, a continuous scale rating the subjective magnitude of problems was more desirable than a categorical variable, and we included well-validated instruments often used in comparable research on post-traumatic symptoms, personality functioning, and personality problems. Nonetheless, self-reports may be more subject to possible confounders, affecting the interpretation of items or willingness to respond (120, 121). Moreover, assessment of PD was performed by trained clinicians using validated and systematically structured diagnostic interviews. As demonstrated in **Table 3**, diagnostic observer-based evaluation of PD largely corresponded to patient self-report of personality functioning (LPFS > cutoff: 81%, any PD: 79%).

The cross-sectional design of the study limits inferences. Temporal ambiguity inherent in this design limits conclusions to be drawn from the mediation analyses in terms of causal mechanisms and direction of associations.

## Conclusion

The present study is one of the few clinical studies with a dimensional focus on SH severity, the magnitude of post-traumatic symptom burden, the level of personality functioning, and direct and indirect associations between these aspects. The study demonstrates that SH severity was significantly and independently associated with higher post-traumatic symptom burden, as well as with greater impairment in self domains of personality functioning. The results demonstrate how these aspects together represent a common and complex cluster of symptoms among patients with repetitive, severe SH. The study further demonstrates that the association between SH severity and post-traumatic symptom burden was partially mediated by personality functioning capacities within the self domains. These findings suggest a profound role of personality functioning in the development of severe SH and thus support broad clinical evaluation of both traumatic burden and personality functioning capacities when encountering patients with SH. Treatment for patients with SH specifically addressing self-destructive actions and trauma management in relation to self-aspects of personality functioning may help prevent severe SH development.

## Data Availability

The datasets presented in this article are not readily available due to concerns of confidentiality. Requests to access the datasets should be directed to e.h.kvarstein@medisin.uio.no.
