# Supplementary material for: Possible drivers of self-harm severity - relative contributions of post-traumatic stress and levels of personality functioning – results from the Extreme Challenges project
Source: Front Psychiatry. 2026 Sep 21;17:1911977. doi: 10.3389/fpsyt.2026.1911977 (PMC13635847; doi:10.3389/fpsyt.2026.1911977)
Supplement: Supplementary file 1 [file DataSheet1.pdf]

Supplementary file

Supplementary Table 1: Correlations between key variables

		SH severity	PCL-5 sum	T scores SIPP-SF domains					LPFS-BF domains	
				Self control	Social Concordance	Identity Integration	Relational functioning	Responsibility	Self	Interpersonal
SH severity	Pearson Correlation	1	,282**	-,270**	-,095	-,294**	-,086	-,082	,198**	,071
	Sig. (2-tailed)		<,001	<,001	,056	<,001	,086	,101	<,001	,153
	N	406	404	405	405	405	405	405	404	404
PCL-5 sumscore	Pearson Correlation	,282**	1	-,291**	-,174**	-,265**	-,117*	-,083	,277**	,192**
	Sig. (2-tailed)	<,001		<,001	<,001	<,001	,018	,094	<,001	<,001
	N	404	406	405	405	405	405	405	406	406
T-SIPP-SF self control	Pearson Correlation	-,270**	-,291**	1	,693**	,472**	,286**	,449**	-,526**	-,584**
	Sig. (2-tailed)	<,001	<,001		<,001	<,001	<,001	<,001	<,001	<,001
	N	405	405	407	407	407	407	407	405	405
T-SIPP-SF Social concordance	Pearson Correlation	-,095	-,174**	,693**	1	,321**	,361**	,488**	-,351**	-,693**
	Sig. (2-tailed)	,056	<,001	<,001		<,001	<,001	<,001	<,001	<,001
	N	405	405	407	407	407	407	407	405	405
T-SIPP-SF Identity Integration	Pearson Correlation	-,294**	-,265**	,472**	,321**	1	,509**	,344**	-,682**	-,348**
	Sig. (2-tailed)	<,001	<,001	<,001	<,001		<,001	<,001	<,001	<,001
	N	405	405	407	407	407	407	407	405	405
T-SIPP-SF Relational functioning	Pearson Correlation	-,086	-,117*	,286**	,361**	,509**	1	,305**	-,429**	-,438**
	Sig. (2-tailed)	,086	,018	<,001	<,001	<,001		<,001	<,001	<,001
	N	405	405	407	407	407	407	407	405	405
T-SIPP-SF Responsibility	Pearson Correlation	-,082	-,083	,449**	,488**	,344**	,305**	1	-,382**	-,459**
	Sig. (2-tailed)	,101	,094	<,001	<,001	<,001	<,001		<,001	<,001
	N	405	405	407	407	407	407	407	405	405
LPFS-BF Self domain	Pearson Correlation	,198**	,277**	-,526**	-,351**	-,682**	-,429**	-,382**	1	,485**
	Sig. (2-tailed)	<,001	<,001	<,001	<,001	<,001	<,001	<,001		<,001
	N	404	406	405	405	405	405	405	406	406
LPFS-BF Interpersonal domain	Pearson Correlation	,071	,192**	-,584**	-,693**	-,348**	-,438**	-,459**	,485**	1
	Sig. (2-tailed)	,153	<,001	<,001	<,001	<,001	<,001	<,001	<,001	
	N	404	406	405	405	405	405	405	406	406

**. Correlation is significant at the 0.01 level (2-tailed).
*. Correlation is significant at the 0.05 level (2-tailed).

Figure 1 Mediation model

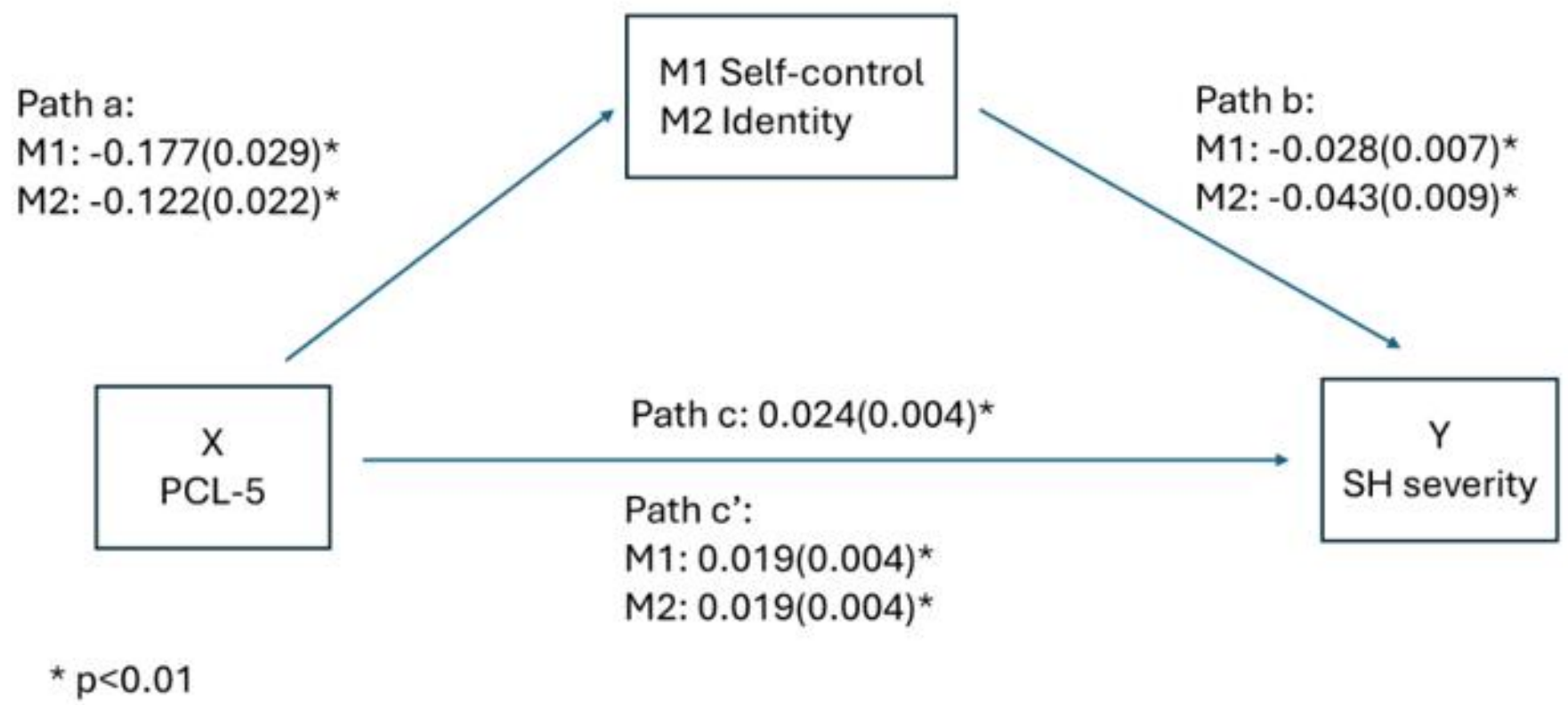

Figure 1 illustrates the mediation models and presents unstandardized beta coefficients with standard errors for each pathway.
